# TP-0903 Is Active in Preclinical Models of Acute Myeloid Leukemia with *TP53* Mutation/Deletion

**DOI:** 10.3390/cancers15010029

**Published:** 2022-12-21

**Authors:** Eric D. Eisenmann, Jack C. Stromatt, Sydney Fobare, Kevin M. Huang, Daelynn R. Buelow, Shelley Orwick, Jae Yoon Jeon, Robert H. Weber, Bill Larsen, Alice S. Mims, Erin Hertlein, John C. Byrd, Sharyn D. Baker

**Affiliations:** 1 Division of Pharmaceutics and Pharmacology, The Ohio State University, Columbus, OH 43212, USA; 2 Division of Hematology, The Ohio State University, Columbus, OH 43212, USA; 3 Department of Internal Medicine, University of Cincinnati, Cincinnati, OH 45267, USA

**Keywords:** *TP53*, AML, TP-0903, decitabine, multikinase, aurora kinase

## Abstract

**Simple Summary:**

Acute myeloid leukemia (AML) with mutations in the tumor suppressor gene *TP53* is rapidly lethal for most patients. Here, we investigated the preclinical activity of TP-0903, a multikinase inhibitor that inhibits kinases with potential synthetical lethality in *TP53* mutant AML. TP-0903 inhibited cell viability and induced apoptosis in multiple *TP53* mutant AML cell lines at nanomolar concentrations in vitro. TP-0903, both alone and in combination with decitabine, the current standard of care, improved survival in two xenograft models of *TP53* mutant AML. These results demonstrate that TP-0903 has activity in AML with *TP53* dysfunction and support the clinical evaluation of TP-0903 in combination with decitabine in *TP53* mutant AML.

**Abstract:**

Acute myeloid leukemia (AML) with mutations in the tumor suppressor gene *TP53* confers a dismal prognosis with 3-year overall survival of <5%. While inhibition of kinases involved in cell cycle regulation induces synthetic lethality in a variety of *TP53* mutant cancers, this strategy has not been evaluated in mutant *TP53* AML. Previously, we demonstrated that TP-0903 is a novel multikinase inhibitor with low nM activity against AURKA/B, Chk1/2, and other cell cycle regulators. Here, we evaluated the preclinical activity of TP-0903 in *TP53* mutant AML cell lines, including a single-cell clone of MV4-11 containing a *TP53* mutation (R248W), Kasumi-1 (R248Q), and HL-60 (*TP 53* null). TP-0903 inhibited cell viability (IC50, 12–32 nM) and induced apoptosis at 50 nM. By immunoblot, 50 nM TP-0903 upregulated pChk1/2 and pH2AX, suggesting induction of DNA damage. The combination of TP-0903 and decitabine was additive in vitro, and in vivo significantly prolonged median survival compared to single-agent treatments in mice xenografted with HL-60 (vehicle, 46 days; decitabine, 55 days; TP-0903, 63 days; combination, 75 days) or MV4-11 (R248W) (51 days; 62 days; 81 days; 89 days) (*p* < 0.001). Together, these results provide scientific premise for the clinical evaluation of TP-0903 in combination with decitabine in *TP53* mutant AML.

## 1. Introduction

Acute myeloid leukemia (AML) is a deadly hematological malignancy typified by heterogeneous cytogenetic abnormalities and genetic mutations [1]. Despite being present in only <10% of de novo AML cases among younger patients [2], mutant *TP53* is present in up to 30% of treatment-related AML cases [3] and ~60% of AML cases with complex karyotype (CK) (i.e., AML containing ≥3 genetic aberrations) [2,3] and 20% of elderly patients [4]. Despite advances in the overall treatment of AML [1], mutations in the tumor suppressor gene *TP53* continue to confer a particularly dismal prognosis with a 3-year overall survival rate <5% [5]. The current standard of care for this AML subtype includes treatment with a hypomethylating agent (HMA) such as decitabine or azacytidine [6]. Nonetheless, the median overall survival of *TP53* mutant AML patients treated with decitabine remains <1 year [7]; the addition of venetoclax, a promising inhibitor of the antiapoptotic BCL-2 protein, did not improve decitabine treatment [8]. New treatment approaches are urgently needed to improve the survival of AML patients with mutant *TP53*.

*TP53* is a tumor suppressor gene that encodes for p53, a transcription factor that regulates the cell cycle and acts as a tumor suppressor in response to DNA damage. The DNA damage response (DDR), controlled by p53 and other checkpoint proteins, including the aurora and checkpoint kinases (e.g., Chk1 and Chk2) [9], acts by regulating checkpoints during the cell cycle to facilitate DNA repair; this regulation includes the G1 checkpoint, regulating the cell’s entry into the S phase, and the G2 checkpoint, regulating entry into mitosis. Cells with mutant or deleted *TP53* frequently have a defective G1 checkpoint and are more dependent on the G2 checkpoint to repair DNA damage; the G2 checkpoint allows p53-deficient AML cells to repair genetic lesions and continue through the cell cycle [10,11]. Consistent with this finding, inhibition of kinases involved in the G2 checkpoint, such as aurora kinase A (AURKA) [12], aurora kinase B (AURKB) [13,14], and checkpoint kinase 1 (Chk1) [15], has induced mitotic catastrophe and p53-independent cell death in *TP53* mutant cancer cells. Inhibitors of each of these cell cycle kinases are in different stages of clinical development for the treatment of AML [9,16]. However, while the combined inhibition of both AURK and Chk has been shown to induce synthetic lethality in ovarian cancer [17], a type of cancer characterized by frequent *TP53* mutations, this strategy has not been evaluated in *TP53* mutant AML.

We previously demonstrated that TP-0903, a small molecule originally developed as an AXL inhibitor [18], is a multikinase inhibitor with low nM activity against AURKA/B, Chk1/2, and other cell cycle regulators and has activity in models of drug-resistant acute myeloid leukemia with wildtype *TP53* [19]. TP-0903 inhibits kinases previously shown to induce synthetic lethality in cancers with frequent *TP53* mutations, including invasive breast cancer [20,21] and pancreatic cancer [22]. Together, these data provided the scientific rationale to evaluate TP-0903 activity in *TP53* mutant AML.

## 2. Materials and Methods

### 2.1. Chemical and Reagents

Antibodies against p53 (48818; clone DO-7), p21 (2947; clone 12D1), pH2AX (Ser 139) (80312; clone D7T2V), pH2AX (Ser139/Tyr142) (5438; clone N/A), H2AX (7631; clone D17A3), pAURKA(Thr288)/B(Thr232)/C(Thr198) (2914; clone D13A11), AURKA (91590; clone D3V7T), AURKB (3094; clone N/A), pChk1 (Ser345) (2348; clone 133D3), pChk2 (Thr68) (2197; clone C13C1), Chk1 (2360; clone 2G1D5), Chk2 (6334; clone D9C6), vinculin (13901; clone E1E9V), GAPDH (5174S; clone D16H11), and HRP-conjugate secondary anti-rabbit (7074) were obtained from Cell Signaling Technology (CST) (Danvers, MA, USA). An antibody against MDM2 (MA1-24755) and qPCR primers for p21 (Hs00355782) and PUMA (Hs00248075) were obtained from ThermoFisher (Waltham, MA, USA). An antibody against pH2AX (Ser139) (ab81299) for immunohistochemistry was obtained from Abcam (Waltham, MA, USA). ImmPRESS Horse Anti-Rabbit (MP-7401), a secondary antibody for immunohistochemistry, was obtained from Vector Laboratories (Newark, CA, USA). Drugs were obtained from the following sources: TP-0903 tartrate (SPD Oncology, Cambridge, MA, USA), decitabine (LC-Labs, Woburn, MA, USA), and RG7388 (MedChemExpress, Monmouth Junction, NJ, USA). 

### 2.2. Cell Culture

MV4-11 (DSMZ; Braunschweig, Germany), Kasumi-1 (acquired from Dr. Ramiro Garzon’s lab, The Ohio State University, Columbus, OH, USA), and HL-60 (ATCC) cells were obtained and maintained in RPMI (Thermo Fisher Scientific) with 10% FBS. Cell lines were verified using short tandem repeat (STR) profiling. Cells were used within 30 passages after thawing and were routinely checked to ensure there was no mycoplasma contamination (MycoAlert Detection Kit, Lonza).

### 2.3. Generation of the MV4-11 (R248W) Cells and Mutational Analysis

MV4-11 cells received from DSMZ harbor a low frequency TP53 mutation [23]. To generate a clone with *TP53* mutant MV4-11, individual cells were sorted into 96-well plates using the FACSAria Fusion Flow Cytometer (BD Biosciences, Franklin Lakes, NJ, USA). DNA was isolated from clones that grew out using the QIAamp DNA Mini Kit (QIAGEN, Hilden, Germany). We confirmed the clones were MV4-11 cells using STR analysis in the OSU Comprehensive Cancer Center (OSUCCC) Genomics Shared Resource. Clones were screened for the presence of an R248W *TP53* mutation using Sanger sequencing at the OSUCCC Genomics Shared Resource and analyzed with Sequencher (Gene Code, Ann Arbor, MI, USA). Primers used for sequencing TP53 were 5′-CCTCATCTTGGGCCTGTGTT-3′ (forward) and 5′-AGTGTGCAGGGTGGCAAGTG-3′ (reverse). A *TP53* mutant and wild-type (WT) clone were selected for further sequencing using a 80-gene capture panel to look for other common AML mutations [24]. Genomic DNA fragmentation was done via focused ultrasonication (Covaris, Woburn, MA, USA). NGS libraries were prepared using a KAPA HyperPlus Kit (Roche, Pleasanton, CA, USA). Target enrichment was performed with xGen Lockdown Probes (IDT, Coralville, IA, USA). Libraries were sequenced using the Illumina HiSeq 4000 (Illumina, San Diego, CA, USA). The Burrows-Wheeler Aligner (BWA) was used to align sequenced reads to the hg19 genome build. UMI-consensus calling on the aligned reads was performed using Picard Tools. The Genome Analysis Toolkit (GATK) was used to realign insertions and deletions in the aligned reads and to perform base quality score recalibration for those realigned regions. Variant calling was performed using GATK’s MuTect2. After variant calling, variants were annotated using SnpEff and vcfanno along with the dbsnp, COSMIC, 1000 genomes, and 6500 exomes variant databases. The Mucor3 algorithm was the baseline for integrative mutation assessment. The Integrative Genomics Viewer was used to visually inspect the aligned reads of all called variants underwent. Variants that were annotated as a likely germline variant by 1000 genomes or 6500 exomes and visually suspected of being germline were removed. Variants in regions of high discrepancy, low quality, tandem repeats, or mononucleotide runs were also excluded. The NGS analysis revealed that the *TP53* mutant clone also harbored a *KMT2A* S873R variant; no information about this variant was available in COSMIC or dpSNP. At this point, additional clones were screened from the original single cell sort for the presence of the *TP53* R248W and the absence of the *KMT2A* S873R; however, no clones with this combination were found and work was carried out with the original mutant clone.

### 2.4. Characterization of the MV4-11 TP53 Mutant (R248W) Cells via Gamma Irradiation

MV4-11 *TP53* mutant (R248W) and MV4-11 wild-type (WT) cells were fetal bovine serum starved for 15 h prior to gamma irradiation. Cells were exposed to either 0, 10, or 20 Gray (Gy) of radiation. At 0, 2, 10, and 24 h after irradiation, cells were collected for subsequent RNA and protein isolation. Immunoblots were run to probe for p53, p21, pH2AX, and MDM2. Real-time quantitative PCR analysis was performed to analyze the transcriptional expression of p21 and PUMA using the ViiA 7 Real-Time PCR system. 

### 2.5. Immunoblotting

Cell lines were treated with drugs for 4 h, then cells were lysed with RIPA buffer (CST) supplemented with protease and phosphatase inhibitors. The concentration of each cell lysate was determined by BCA and an equal concentration of protein was separated on Bis-Tris 4–12% SDS-polyacrylamide gels with Invitrogen MOPS buffer (ThermoFisher, Waltham, MA, USA) and transferred to PVDF membranes followed by Western blot analysis, consistent with manufacturer’s instructions. Western blots were developed with either Signal Fire ECL reagent (CST) or SuperSignal West Femto Maximum Sensitivity Substrate (ThermoFisher) using film. Western blots were quantified using ImageJ (U. S. National Institutes of Health, Bethesda, MD, USA) and normalized to respective loading controls. Drug treatments and Western blots were repeated in 2–3 biological replicate experiments.

### 2.6. Kinase Profiling

Inhibition of AURKA/B and Chk1/2 by TP-0903 was determined in a kinase assay (Reaction Biology (Malvern, PA, USA)).

### 2.7. Cell Viability Assessment

Cells lines were treated with drugs for 72 h before treatment effects were assessed by MTT assay (Sigma), as previously described [19]. 100,000 cells/well were plated in a 96-well plate and treated with increasing concentrations of drug. For assays assessing the impact of combining two drugs, surface–response analysis was performed using the Combenefit software (v. 2.0.2, Cancer Research UK Cambridge Institute, Cambridge, UK) [25].

### 2.8. Cell Cycle Analysis 

Asynchronous cells were treated with DMSO or 20 nM TP-0903 for up to 24 h. At indicated time points, cells were collected and washed with 0.1% EDTA PBS (Ricca Chemical Company, Arlington, TX, USA) followed by only PBS. Cells were then fixed on ice with ice-cold 70% ethanol for 30 min. Cells were either stored at −20 °C for up to 1 month or processed immediately. Cells were spun down at 450× *g* for 10 min and stained with DAPI (final concentration 0.1 ug/1 mL) in 0.1% Triton-X (ThermoFisher) PBS for 30 min at room temperature and protected from light. The DNA content was determined using a BD LSR II flow cytometer in the OSUCCC Flow Cytometry Shared Resource. The cell cycle distribution was analyzed using FlowJo (BD Biosciences).

### 2.9. Apoptosis Assessment and CD11b Expression Assays

Cells were treated with DMSO, 20 nM, or 50 nM TP-0903 for up to 48 h. At the indicated time points, cells were collected and washed with PBS. DAPI was added to each sample as a viability dye at a final concentration of 0.1 ug/1 mL. For apoptosis assays, cells were incubated with annexin V-APC (BioLegend, San Diego, CA, USA)) according to the manufacturer’s instructions. For analysis of CD11b, CD11b expression was measured using an anti-CD11b-APC antibody (BioLegend, San Diego, CA, USA). Two-color flow cytometry was performed in the OSUCCC Flow Cytometry Shared Resource, and data were analyzed using FlowJo (BD Biosciences). 

### 2.10. RNA Isolation and RT-PCR

RNA was extracted from cells using Trizol-Cholorform. cDNA was generated from 0.5 µg of RNA using the SuperScript IV First-Strand Synthesis System (ThermoFisher)). Real time PCR for target genes and GAPDH, the housekeeping target, was conducted using the TaqMan FAST method (ThermoFisher) with 50 ng of cDNA. Target Ct (threshold cycles) was standardized to the GAPDH Ct and graphed as 2^−ΔCt^.

### 2.11. Growth Curves

Cells were seeded in triplicate at a concentration of 1.5 × 10^5^ cells/mL in 10 mL and were treated with DMSO, 10 nM TP-0903, or 50 nM TP-0903. Cells were counted every 2–3 days using Trypan Blue and normalized to Day 0 counts. 

### 2.12. Murine Xenograft Models

MV4-11 (R248W) cells were transduced with a lentiviral vector containing a YFP/luciferase construct (MV4-11 (R248W)-Luc+) to permit the monitoring of engraftment through bioluminescence imaging. MV4-11 (R248W)-Luc+ were verified as homogeneous *TP53* mutant by Sanger sequencing. Female 8-12 week-old NSG mice were procured from Jackson Laboratories (stock number: 005557; Bar Harbor, Maine, USA) and injected intravenously via tail vein injection (TVI) with one million MV4-11 (R248W)-Luc+ cells (5–6 per treatment cohort). Engraftment and growth of leukemic cells were monitored weekly by noninvasive bioluminescence imaging; after injection of D-luciferin (150 mg/kg i.p.; Gold Biotechnology Inc., St. Louis, MO, USA), mice were assessed with Xenogen IVIS-200 imaging system (Perkin Elmer, WA, MA), as done previously [19]. Mice were randomized to treatment groups based on signal intensity and leukemia burden. Fourteen days after TVI, mice were treated with vehicle, TP-0903 (50 mg/kg orally; 5 days on/2 days off), decitabine (0.2 mg/kg i.p.; 4 days on/10 days off), or the combination for three cycles. TP-0903 was formulated in 5% (*w*/*v*) vitamin E TPGS (Antare Health Products, Jonesborough, TN, USA) and 1% Tween 80 (Sigma-Aldrich). Decitabine was dissolved in PBS. All mice were observed daily and humanely euthanized when showing signs of progressive disease including hind limb paralysis, weight loss of more than 20%, and lethargy. All animal studies were approved by the OSU Institutional Animal Care and Use Committee. 

HL-60 xenografts were conducted by Charles River Laboratories (Ashland, OH, USA). 8–12 week old female NOD-*Prkdc^em26Cd52^Il2rg^em26Cd22^*/NjuCrl (NCG) mice were injected intravenously with HL-60 cells (10 per treatment cohort). Fourteen days after TVI, Mice were treated with vehicle, TP-0903 (50 mg/kg orally; 5 days on/2 days off), decitabine (0.4 mg/kg i.p.; 4 days on/10 days off) or the combination continuously until mice succumbed to leukemia.

### 2.13. Targeted Gene Sequencing 

MV4-11 (WT) and MV4-11 (R248W) cell lines and bone marrow samples collected after mice with MV4-11 (R248W)-Luc+ xenografts succumbed to leukemia and were analyzed by targeted gene sequencing of 80 genes, as previously described [24]. MV4-11 (WT) and MV4-11 (R248W) were collected at both early and late passages. Bone marrow samples were collected from mice with MV4-11 (R248W)-Luc+ xenografts treated with vehicle, decitabine, TP-0903, or the combination. Samples were pooled and analyzed on a MiSeq system using the Illuminia MiSeq Reagent Kit v3. Sequencing was performed in the OSUCCC Genomics Shared Resource. Illumina Isis Banded Smith Waterman aligner and hg19 genome were used to align the sequences. Single nucleotide variant (SNV) and indel calling were performed using MuTect [26] and Varscan2. A variant allele fraction (VAF) cut-off of 0.10 was set for reporting mutations. SNV that are reported as pathogenic SNP were considered mutations. All other SNV needed to be absent from 1000 Genome database, dsSNP137 or dsSNP142. Visual inspection of all variants was carried out using Integrative Genomics Viewer (Broad Institute Cambridge, MA, USA).

### 2.14. Immunohistochemistry

Immunohistochemistry was completed in collaboration with the OSUCCC Comparative Pathology and Digital Imaging Shared Resource. After 4 h of drug treatment, cells were pelleted and fixed for 48 h in 10% neutral buffered formalin. Paraffin processing was completed using standard techniques and slides were stained for pH2AX or the appropriate control. Representative photomicrographs were taken using a Nikon Eclipse Ci-L Upright Microscope (Nikon Instruments, Inc., Melville, NY, USA), an 18-megapixel Olympus SC180 microscope-mounted digital camera, and cellSens imaging software (Olympus Life Science, Center Valley, PA, USA).

### 2.15. Statistics

Statistical analyses to compare specific groups were performed with GraphPad Prism software using unpaired two-tailed Student’s *t*-tests without correction for repeated analysis. Survival was depicted using Kaplan–Meier plots and compared using the log-ranked test. A *p* < 0.05 was determined to be statistically significant. Data represent the mean ± standard error of the mean.

## 3. Results

### 3.1. Isolation and Characterization of a TP53 Mutant MV4-11 Clone

To generate an additional in vitro model of *TP53* mutant AML, we isolated and characterized single-cell clones containing mutant (R248W) or wild-type (WT) *TP53* from the established MV4-11 AML cell line, as has been done previously [23]. After isolation and expansion of individual clones, we used sequencing to identify a clone with mutant *TP53* (Figure S1A). Next-generation sequencing performed with MV4-11 (WT) and MV4-11 (R248W) cells collected at early and late passages demonstrated that mutations were stable in each cell line (Supplementary Table S1). Notably, the isolated MV4-11 (R248W) clone also had a mutation in KMT2A (S873R) that was not present in WT cells. Nonetheless, the MV4-11 (R248W) had a defective p53 response. Having identified a mutant clone, we used immunoblotting to compare the regulation of MDM2, p21, and pH2AX and real-time PCR to compare the regulation of p21 and PUMA by the MV4-11 (R248W) and MV4-11 (WT) clones following gamma irradiation (Figure S1B,C). MV4-11 (R248W) cells displayed a defective p53 response; when compared against MV4-11 (WT) cells, MV4-11 (R248W) cells had smaller increases in the expression of p53-regulated genes following gamma irradiation. Further validating this defective p53 response, MV4-11 (R248W) cells were less sensitive to RG7388, an inhibitor of MDM2 [27]; in an MTT assay, MV4-11 (R248W) cells had a >10-fold higher IC50 than MV4-11 (WT) cells. RG7388 induced apoptosis in MV4-11 (WT) cells, but not in MV4-11 (R248W) cells (Figure S1D).

### 3.2. TP-0903 Inhibits Aurora Kinases

**Figure 1:**
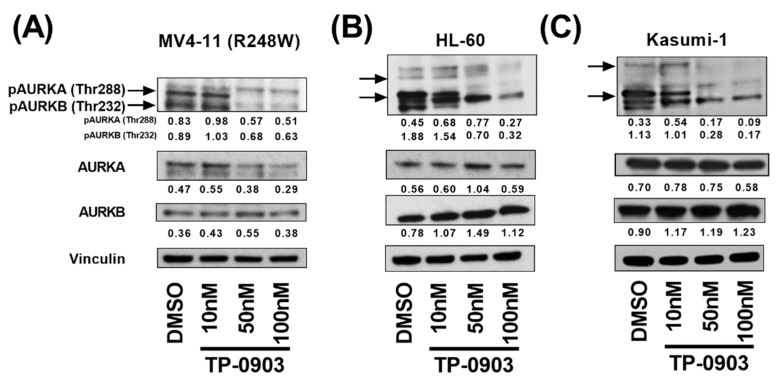
TP-0903 inhibits aurora kinases in *TP53* mutant AML cell lines. (**A**) MV4-11 (R248W) (**B**) HL-60 or (**C**) Kasumi-1 cells were treated with DMSO or increasing concentrations of TP-0903 for 4 h. Western blot analysis was performed on whole-cell lysates run on parallel gels with the indicated antibodies. Vinculin served as the loading control for each lysate. Immunoblots were quantified against respective loading controls using ImageJ. Data are representative of 3 independent experiments.

Previously, we found that TP-0903 inhibited AURKA and AURKB in a binding affinity assay and in cell lines with characteristics of high-risk AML including *FLT3* and *RAS* mutations [19]. Based on these data, we sought to determine whether TP-0903 inhibits AURKA and AURKB in a kinase assay and in *TP53* mutant AML cell lines. In a kinase assay, TP-0903 inhibited AURKA and AURKB with EC50s of 0.66 nM and 2.23 nM, respectively (Figure S2). Based on these data, we determined the ability of TP-0903 to inhibit these kinases in *TP53* mutant AML cell lines including HL-60 (*TP53* null), Kasumi-1 (R248Q), and MV4-11 (R248W) cells. TP-0903 inhibited both pAURKA (Thr 288) and pAURKB (Thr 232), important autophosphorylation sites [28], in each cell line with a robust decrease at 50 nM, an achievable concentration in bone marrow [19] (Figure 1). Based on these data and data suggesting that AURK inhibition is synthetic lethal in *TP53* mutant cancers [29], we sought to determine the in vitro efficacy of TP-0903.

### 3.3. TP-0903 Has In Vitro Activity in TP53 Mutant AML Cell Lines

**Figure 2:**
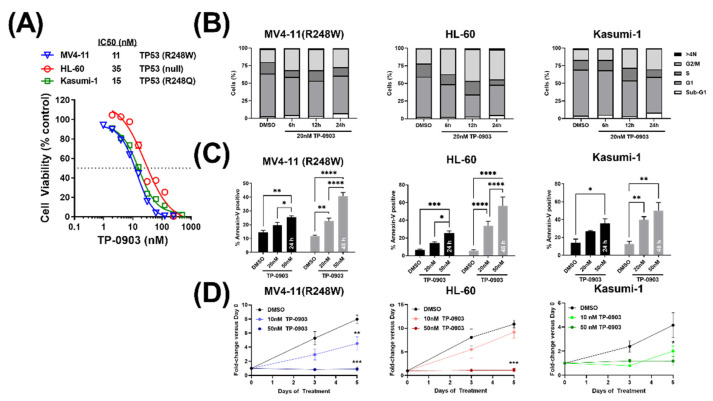
TP-0903 has in vitro activity in TP53 mutant AML cell lines. (**A**) MTT cell viability assay after MV4-11 (R248W), HL-60, and Kasumi-1 cell lines were treated with increasing concentrations of TP-0903 (n = 18 across three independent experiments). (**B**) Cell cycle analysis performed in MV4-11 (R248W), HL-60, and Kasumi-1 cell lines treated with 20 nM TP-0903 determined by flow cytometry using DAPI (n = 4–5). (**C**) Induction of apoptosis in MV4-11 (R248W), HL-60, and Kasumi-1 cell lines treated with TP-0903 (20 nM or 50 nM) for 24 h (black bar) or 48 h (grey bar) determined by flow cytometry using Annexin V (n = 3). Data represent the mean ± SEM. (**D**) MV4-11 (R248W), HL-60, and Kasumi-1 cell lines were plated with increasing concentrations of TP-0903 (10 nM, 50 nM) and counted on days 3 and 5. Cell counts/viability determined by trypan blue exclusion with Nexcelom Cellometer (n = 3). (* *p* < 0.05, ** *p* < 0.01, *** *p* <0.001, **** *p* <0.0001).

First, we assessed the ability of TP-0903 to inhibit the viability of *TP53* mutant AML cells. In an MTT assay, TP-0903 inhibited the viability of MV4-11 (R248W) (IC50: 11 nM ± 0.5 nM (SE)), HL-60 (IC50: 35 nM ± 2.4 nM (SE)), and Kasumi-1 cells (IC50: 15 nM ± 1.1 nM (SE)) (Figure 2A). Then, to determine how TP-0903 reduces cell viability, we performed cell cycle and apoptosis assays in each of these cell lines. Consistent with our previous data [19] and TP-0903’s inhibition of AURKA, TP-0903 induced a G2/M arrest and apoptosis in each of these cell lines (Figure 2B,C). In addition, we observed decreased growth over 5 days in the presence of TP-0903 (Figure 2D). Based on our previous findings demonstrating that TP-0903 induced AML cell differentiation in AML cells with wildtype (WT) *TP53* [19], we performed similar experiments directly comparing our single-cell sorted MV4-11 (WT) and MV4-11 (R248W) cell lines. In both cell lines, TP-0903 increased the expression of lysozyme and GCSFR (Figure S3A) and the cell surface expression of CD11b (Figure S3B), which suggests that TP-0903 induces cellular differentiation in *TP53* mutant cells. Based on the observed G2/M arrest, as well as our prior observation that TP-0903 inhibits Chk kinases in other AML cell types, we anticipated that TP-0903 would induce a DNA damage response in *TP53* mutant AML cell lines. 

### 3.4. TP-0903 Inhibits Checkpoint Kinases

**Figure 3:**
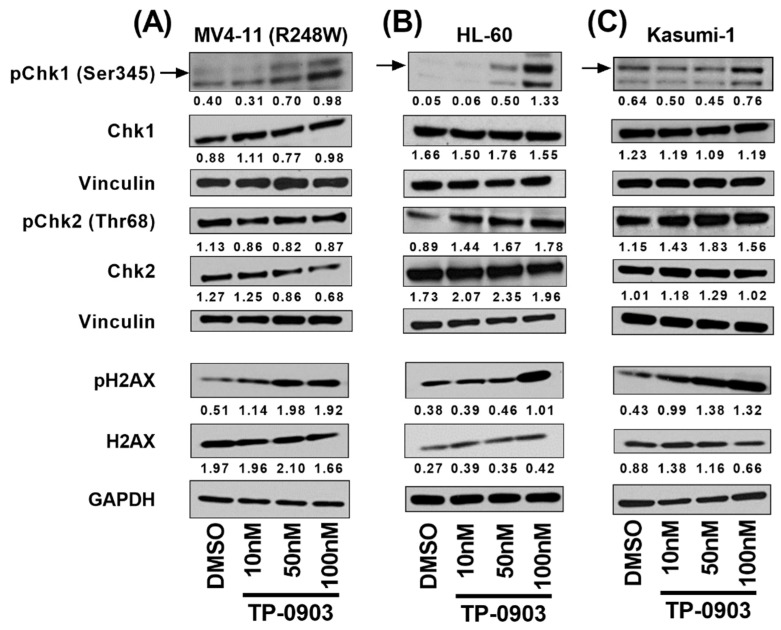
TP-0903 induces DNA damage response in TP53 mutant AML cell lines. (**A**) MV4-11 (R248W), (**B**) HL-60, and (**C**) Kasumi-1 cells were treated with DMSO or TP-0903 at indicated concentrations for 4 h. Immunoblotting was performed to determine the expression of pChk1 (Ser345), pChk2 (Thr68), and pH2AX (Ser139/Tyr142). Vinculin served as the loading control for pChk1/Chk1 and pChk2/Chk2 blots. GAPDH served as the loading control for pH2AX/H2AX blots. Immunoblots were quantified against respective loading controls (vinculin or GAPDH) using ImageJ. Blots are representative of 2–3 independent experiments.

To investigate the hypothesis that inhibition of checkpoint kinases contributes to the efficacy of TP-0903 in *TP53* mutant AML, we first determined the ability of TP-0903 to inhibit these kinases. In a kinase assay, TP-0903 inhibited Chk1 and Chk2 with EC50s of 2.99 nM, and 4.99 nM, respectively (Figure S2). Using our MV4-11 (R248W) cells, we conducted a pilot study and found that pChk1 was substantially upregulated after a 4h treatment with 100 nM TP-0903 (Figure S4). Following this pilot experiment, we determined the effects of TP-0903 on pChk1 and pChk2 in HL-60, Kasumi-1, and MV4-11 (R248W) cell lines. Similar to the pilot study, pChk1, as well as pChk2 were upregulated over a concentration range of TP-0903 from 10 to 100 nM (Figure 3). Consistent with this finding, a prior investigation postulated that as a result of Chk1 inhibition, DNA damage accumulates, which amplifies ATM/ATR signaling, leading to increased phosphorylation of Chk1 (Ser345) and Chk2 (Thr68) [30].

We next investigated the impact of TP-0903 on pH2AX, a marker of DNA damage. In each of our cell lines, TP-0903 treatment led to upregulation of pH2AX by immunoblot, suggesting induction of DNA damage (Figure 3). Consistent with these results, TP-0903 treatment led to substantial upregulation of pH2AX by immunohistochemistry (Figure S5). Consistent with our previous findings in models of high-risk AML with wildtype *TP53* [19], TP-0903 treatment also upregulated pChk1 and pH2AX in MV4-11 (WT) cells (Figures S5 and S6).

### 3.5. Combination of TP-0903 and Decitabine Is Active In Vitro

**Figure 4:**
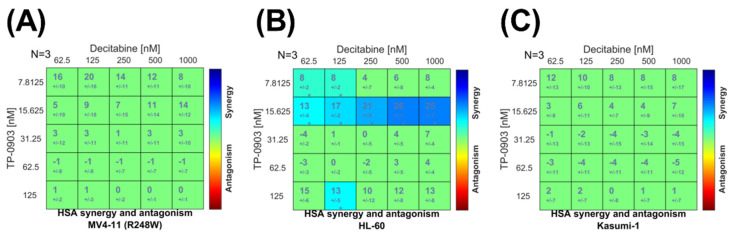
Combinatorial in vitro activity of TP-0903 and decitabine. MTT cell viability assays were performed by treating (**A**) MV4-11 (R248W), (**B**) HL-60, and (**C**) Kasumi-1 cell lines with varying concentrations of TP-0903 and decitabine for 72 h (n = 18 across three independent experiments). Data were analyzed with surface-response using the highest single agent (HSA) model using Combenefit software.

Based on the promising single-agent activity of TP-0903 in vitro, we determined the impact of adding TP-0903 to decitabine, the current standard of care for most cases of *TP53* mutant AML. In an MTT assay, as expected, both TP-0903 and decitabine had single-agent activity and reduced the cell viability of HL-60, Kasumi-1, and MV4-11 (R248W) cells (Figure S7). We then performed combinatorial MTT assays with varying ratios of TP-0903 and decitabine analyzed utilizing response surface techniques [25]. This analysis demonstrated a positive additive distribution (Figure 4).

### 3.6. TP-0903 and Decitabine Improve Survival In Vivo in an HL-60 Xenograft Model

**Figure 5:**
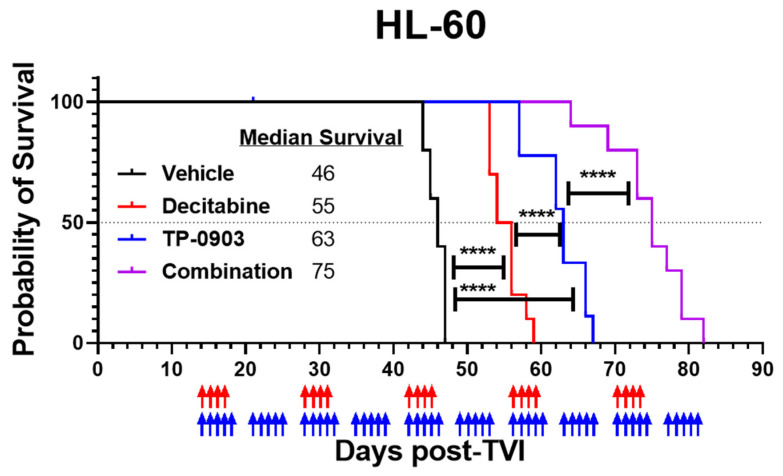
TP-0903 is active in an HL-60 xenograft model. Kaplan–Meier survival curve of NCG mice after intravenous injection with HL-60 cells (10 per treatment cohort). Fourteen days after TVI, mice were treated with vehicle, TP-0903 (50 mg/kg orally; 5 days on/2 days off), decitabine (0.4 mg/kg i.p.; 4 days on/10 days off), or the combination. Blue and red arrows represent days of TP-0903 and decitabine treatment, respectively. The TP-0903/decitabine combination prolonged median survival (75 days) compared to cohorts of mice treated with TP-0903 (63 days), decitabine (55 days), or vehicle (46 days) (**** *p* < 0.0001).

With positive combinatorial in vitro data, we next evaluated the in vivo activity of TP-0903, decitabine, or the combination in an HL-60 xenograft model. Fourteen days after injection with HL-60 cells, mice were treated with vehicle, TP-0903, decitabine, or the combination. The TP-0903/decitabine combination prolonged median survival (75 days) compared to cohorts of mice treated with TP-0903 (63 days), decitabine (55 days), or vehicle (46 days) (*p* < 0.0001) (Figure 5).

### 3.7. TP-0903 Is Active in a MV4-11 (R248W)-Luc+ Xenograft Model

**Figure 6:**
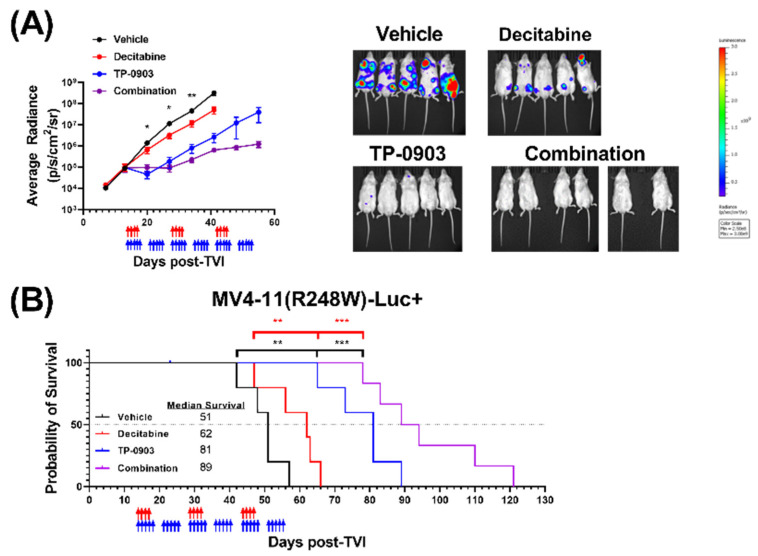
TP-0903 is active in a MV4-11 (R248W)-Luc+ xenograft model. NSG mice were injected intravenously with MV4-11 (R248W) cells transfected with luciferase (MV4-11 (R248W)-Luc+) (5–6 per treatment cohort). Fourteen days after TVI, mice were treated with vehicle, TP-0903 (50 mg/kg orally; 5 days on/2 days off), decitabine (0.2 mg/kg i.p.; 4 days on/10 days off), or the combination for three cycles. Blue and red arrows represent days of TP-0903 and decitabine treatment, respectively. (**A**) Average radiance as determined by whole-body bioluminescence imaging performed weekly (left). Data represent the mean ± SEM. A two-way ANOVA demonstrated a significant difference between groups treated with TP-0903 or vehicle (* *p* < 0.05, ** *p* < 0.01). Representative whole-body bioluminescence images from day 41 (right). Each image uses the same scale. (**B**) Kaplan–Meier survival curve. Survival analysis demonstrated a significant survival advantage in mice treated with TP-0903 alone or the combination versus decitabine alone (** *p* < 0.01, *** *p* < 0.001, respectively) or vehicle (** *p* < 0.01, *** *p* < 0.001).

Based on our positive single-agent and combinatorial in vivo data in the HL-60 mouse model, we next evaluated the activity of TP-0903, decitabine, or the combination in an MV4-11 (R248W)-Luc+ xenograft model. TP-0903, decitabine, and the combination suppressed leukemia outgrowth, as assessed by bioluminescence imagining (Figure 6A), and improved survival (Figure 6B). When compared against the median survival of mice treated with vehicle (51 days) or decitabine (62 days), the survival of mice treated with TP-0903 (81 days) or the combination (89 days) was significantly longer (** *p* < 0.01, *** *p* <0.001., respectively). Next-generation DNA sequencing performed with bone marrow samples collected from one mouse from each treatment group at study endpoint demonstrated that the mutations observed in the MV4-11 (R248W)-Luc+ cells were the same as in the MV4-11 (R248W) cells (Supplementary Table S2). However, the *TP53* mutation had a loss of heterozygosity in all four treatment groups, consistent with mutant p53 stabilization and progression of cancer, as has been observed previously in vivo [31,32].

## 4. Discussion

*TP53* mutant AML patients represent a cohort with a particularly poor prognosis and lacking disease-modifying therapy [33]. While AML-related *TP53* mutations differ in VAF, mutational site, and functional impact, most of these mutations are missense mutations in cancer-associated *TP53* hotspots (e.g., codons 245, 248, and 273) [34], which are represented by our Kasumi-1 (R248Q) and MV4-11 (R248W) cell lines. Nonetheless, it is postulated that both loss of *TP53* (e.g., HL-60 cells) and missense mutations function similarly by preventing or redirecting p53 protein’s DNA binding and downstream activity [34]. Despite a growing appreciation for *TP53* mutations that confer novel functions that could worsen AML (e.g., gain of function (GOF) mutations), there was no difference in survival whether a patient had *TP53* mutations classified as disruptive or non-disruptive [35]. Altogether, despite the ongoing characterization of unique AML-related *TP53* mutations, most *TP53* abnormalities appear to confer a worse prognosis for AML patients; disease-modifying therapy is clearly needed for this patient population. Several novel drugs are under investigation to meet this urgent need [36]. Among these, eprenetapopt (APR-246) likely represents the most promising therapy targeting mutant p53. However, despite promising preclinical data [37], clinical trials have thus far yielded relatively disappointing results, with median overall survival < 1 year [38]. Overall, it remains clear that effective options for this subtype of AML remain lacking. 

Our present findings demonstrating that TP-0903 is effective in vitro and in vivo among several preclinical models of *TP53* mutant AML are consistent with our previous investigation that found TP-0903 to be effective in other high-risk AML subtypes, including AML with mutations in *NRAS* or *FLT3*. Indeed, TP-0903 had nearly equipotent activity in MV4-11 (R248W) cells here as it had in MV4-11 (WT) cells [19]. Given the inhibition of kinases broadly important to AML cells (e.g., FLT3, NRAS, AURKA/B, Chk1/2), it is not surprising that TP-0903 has activity across several different AML subtypes. In fact, this broad activity is particularly appealing for *TP53* mutant AML, which frequently occurs in complex karyotype AML. In this context, it is worth noting that transcriptome analysis implicated FLT3 as a resistance mechanism to APR-246 [39], which is not an anticipated issue with TP-0903 [19]. However, the relative importance of individual kinases to the survival of AML cells in each of our models remains unclear. Future investigations will require elegant experiments to characterize the exact mechanisms underlying TP-0903 cytotoxicity. Nonetheless, here we have demonstrated the potential contribution of inhibition of cell cycle kinases, including AURKs and Chks, to TP-0903 activity. Together, our results suggest that TP-0903 causes DNA damage, amplifying ATM/ATR signaling, leading to increased phosphorylation of Chk1 (Ser345) and H2AX [30] and subsequent cell death.

TP-0903 was effective in combination with decitabine, the current standard of care for *TP53* mutant AML. While cytotoxic concentrations of decitabine cause a G2/M arrest in cancer cells [40,41], it is unclear if concentrations of decitabine attained with lower doses achieve similar outcomes [42,43]. Nonetheless, the addition of TP-0903 to decitabine treatment enhanced drug activity in both in vitro and in vivo preclinical evaluation. Consistent with these results, decitabine has previously been shown to be synergistic with other multikinase inhibitors [44,45]. While the exact mechanisms underlying this synergy are unclear, it is worth noting that, when decitabine was combined with vorinostat, an HDAC inhibitor, the AXL signaling pathway (i.e., TP-0903’s original target) was upregulated and identified as a potential combinatorial target. Altogether, TP-0903 inhibits putative targets in AML and, based on our data, has great potential to be highly effective in this disease state. When mice are administered 40–60 mg/kg TP-0903, plasma exposure of active moieties is in the range observed in patients enrolled to ongoing phase I trials (NCT02729298), and comparable to effective in vitro concentrations used in the present study [19]. Alleviating concerns that this broad activity could be overtly toxic, TP-0903 was acceptably tolerated in first-in-human studies in patients with advanced solid tumors [46]. Consistent with these findings, we’ve previously demonstrated an acceptable broad therapeutic index when comparing concentrations necessary to kill cancer cells (~20 nM) versus those necessary to impair colony formation by healthy bone marrow cells (>100 nM) or the viability of fibroblasts (>1 μM) [19]. 

## 5. Conclusions

Overall, our findings demonstrate that TP-0903 has preclinical activity in *TP53* mutant AML, with enhanced activity when given in combination with decitabine. Together, these results provide scientific premise to evaluate TP-0903 activity in *TP53* mutant AML.

## Data Availability

The data presented in this study are available on request from the corresponding author.

## Supplementary Materials

The following supporting information can be downloaded at: https://www.mdpi.com/article/10.3390/cancers15010029/s1, Table S1. Variant allele frequencies from next-generation sequencing of single-cell clones of MV4-11 wildtype (WT) and MV4-11 TP53 mutant (R248W) cells; Table S2. Variant allele frequencies from next-generation sequencing of bone marrow samples collected from mice that succumbed to leukemia after injection with MV4-11 (R248W)-Luc+ cells and treated with vehicle, TP-0903, decitabine, or the combination; Figure S1. Characterization of MV4-11 (R248W); Figure S2. TP-0903 inhibits AURKA, AURKB, Chk1 and Chk2 in kinase assays; Figure S3. Induction of differentiation by TP-0903 in MV4-11 wild-type (WT) and TP53 mutant (R248W) cells; Figure S4. Effects of TP-0903 on pChk1 in *TP53* mutant MV4-11 cells; Figure S5. Immunohistochemistry demonstrating upregulation of pH2AX with TP-0903 treatment; Figure S6. TP-0903 upregulates pChk kinase and pH2AX in MV4-11 cells; Figure S7. In vitro activity of TP-0903 or decitabine in combinatorial assays.
