# Supplementary material for: TP-0903 Is Active in Preclinical Models of Acute Myeloid Leukemia with TP53 Mutation/Deletion
Source: Cancers (Basel). 2022 Dec 21;15(1):29. doi: 10.3390/cancers15010029 (PMC9817780; doi:10.3390/cancers15010029)
Supplement: Supplementary file 1 [file cancers-15-00029-s001.zip › cancers-2105681-supplementary.pdf]

cancers

MDPI

Supplementary Materials

TP-0903 Is Active in Preclinical Models of Acute Myeloid Leukemia with *TP53* Mutation/Deletion

Eric D. Eisenmann, Jack C. Stromatt, Sydney Fobare, Kevin M. Huang, Daelynn R. Buelow, Shelley Orwick, Jae Yoon Jeon, Robert H. Weber, Bill Larsen, Alice S. Mims, Erin Hertlein, John C. Byrd and Sharyn D. Baker

Table S1. Variant allele frequencies from next-generation sequencing of single-cell clones of MV4-11 wildtype (WT) and MV4-11 *TP53* mutant (R248W) cells.

Gene Name	Amino Acid Change	MV4-11 (WT) Early Passage	MV4-11 (WT) Late Passage	MV4-11 (R248W) Early Passage	MV4-11 (R248W) Late Passage
FLT3	ITD	Present	Present	Present	Present
ATG2B	Cys1847Arg	0.476	0.455	0.483	0.476
TP53	Arg248Trp			0.5	0.426
KMT2A	Ser873Arg			0.558	0.504

Table S2. Variant allele frequencies from next-generation sequencing of bone marrow samples collected from mice that succumbed to leukemia after injection with MV4-11 (R248W)-Luc+ cells and treated with vehicle, TP-0903, decitabine, or the combination.

Gene Name	Amino Acid Change	MV4-11 (R248W)-Luc+ Vehicle	MV4-11 (R248W)-Luc+ TP-0903	MV4-11 (R248W)-Luc+ Decitabine	MV4-11 (R248W)-Luc+ Combination
FLT3	ITD	Present	Present	Present	Present
ATG2B	Cys1847Arg	0.473	0.474	0.47	0.476
TP53	Arg248Trp	1	1	0.999	1
KMT2A	Ser873Arg	0.503	0.51	0.523	0.507

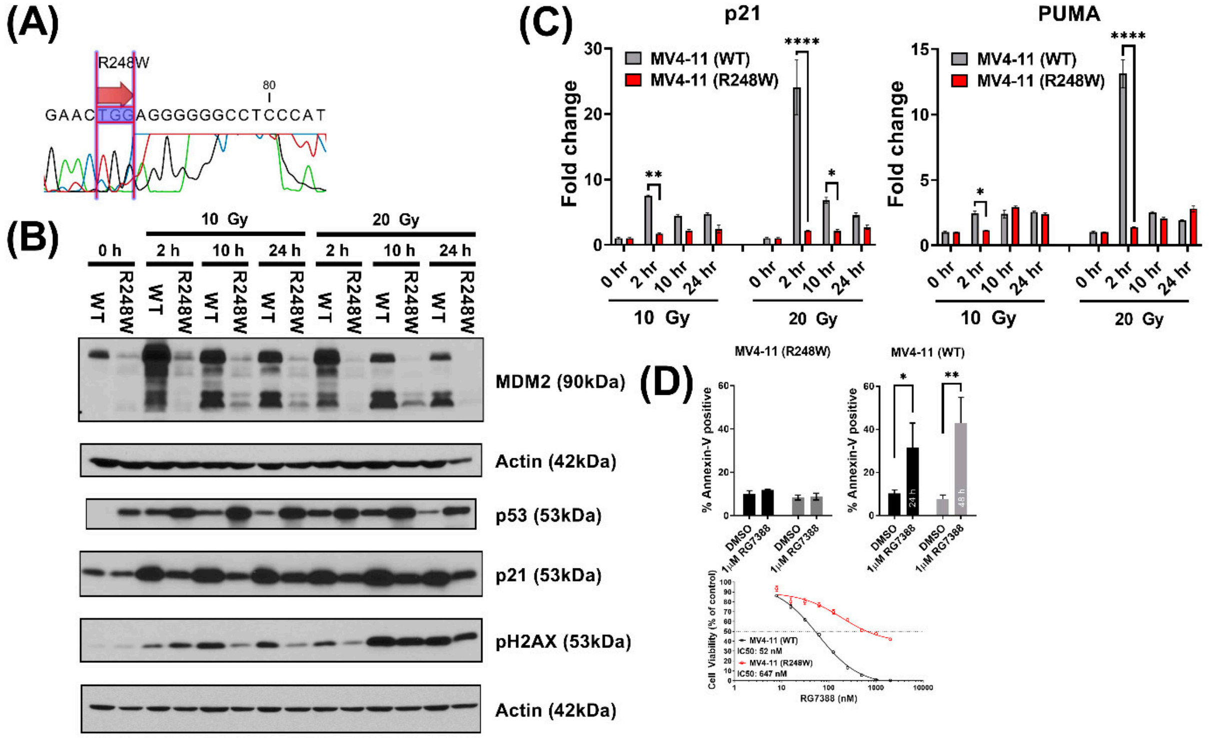

Figure S1. Characterization of MV4-11 (R248W). **(A)** Sequencing demonstrates an R248W mutation in clone isolated from MV4-11 cell line. Irradiated MV4-11 (R248W) and MV4-11 (WT) cells demonstrate defective p53 signaling in MV4-11 (R248W) cells by **(B)** immunoblotting and **(C)** qPCR. **(D)** MV4-11 (R248W) cells are much less sensitive to RG7388, an inhibitor of MDM2, than MV4-11 (WT) cells in apoptosis assays (determined by flow cytometry using Annexin V ($n = 3$)) (top) or MTT cell viability assays ($n = 18$ across three independent experiments) (bottom). *$p < 0.05$, **$p < 0.01$, ****$p < 0.0001$.

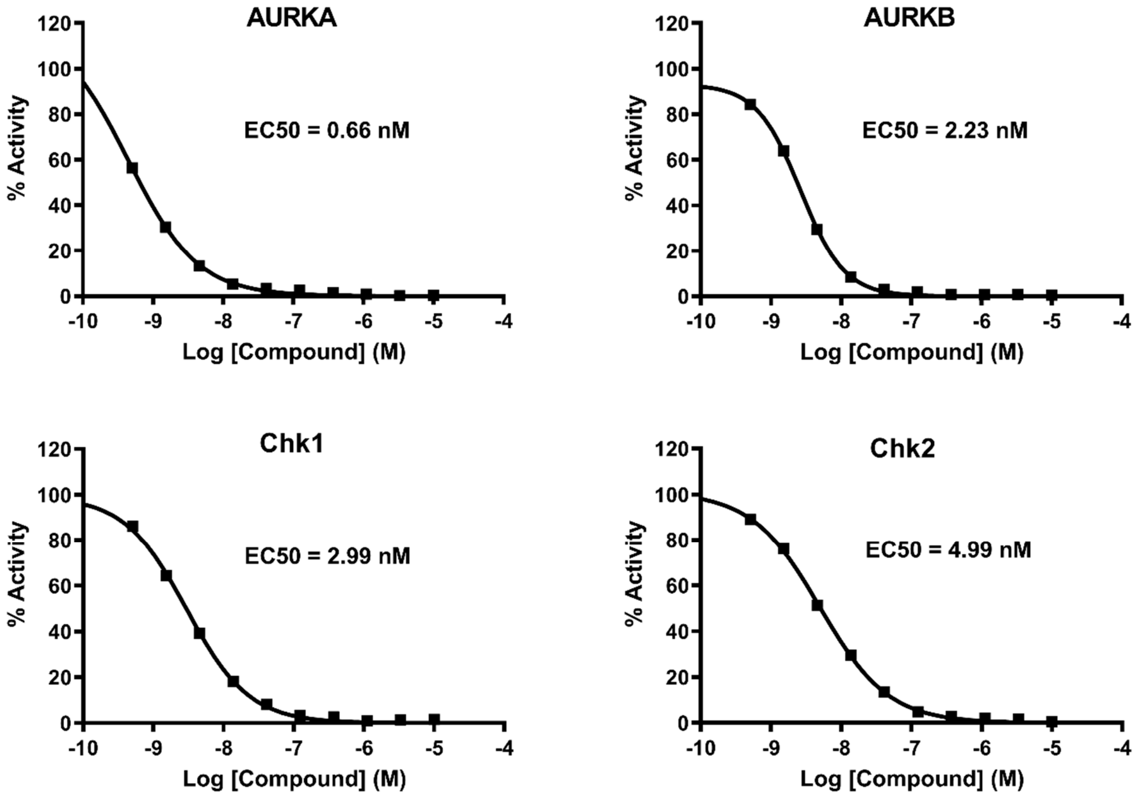

Figure S2. TP-0903 inhibits AURKA, AURKB, Chk1 and Chk2 in kinase assays.

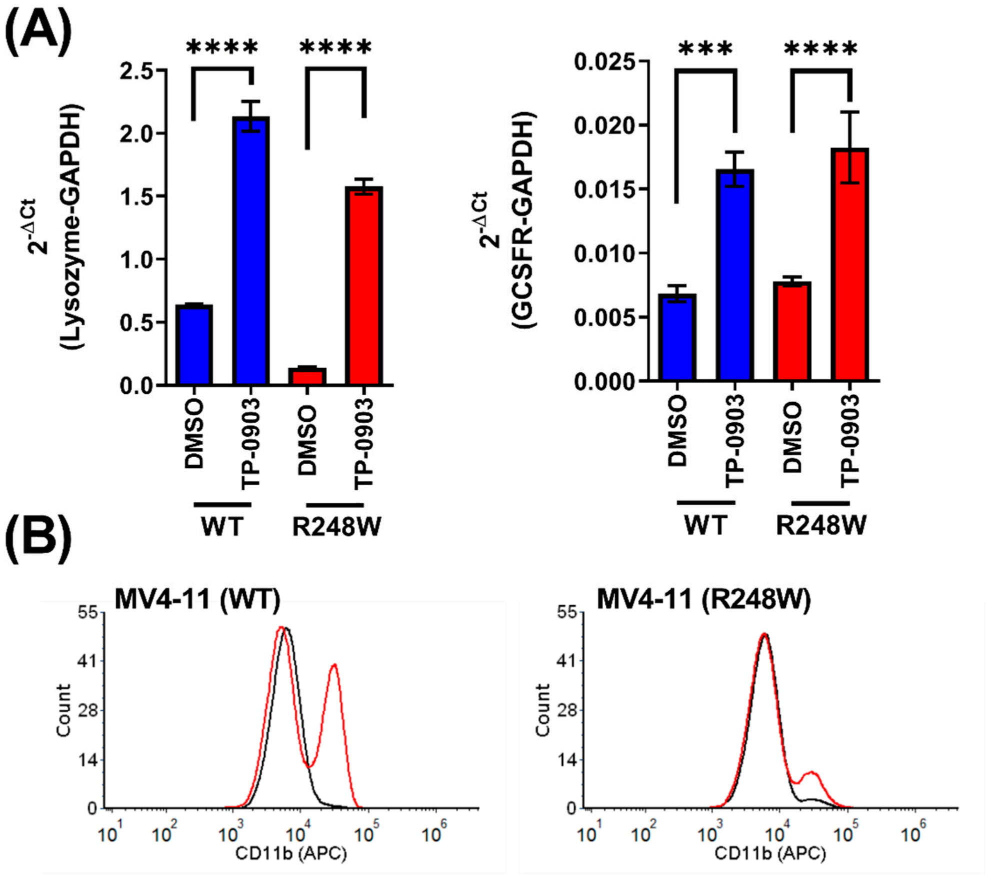

Figure S3. Induction of differentiation by TP-0903 in MV4-11 wild-type (WT) and *TP53* mutant (R248W) cells. **(A)** GCSFR and lysozyme gene expression increases after 72h of TP-0903 treatment ($n = 3$) by RT-PCR. **(B)** Cell differentiation after treatment of MV4-11 (WT) or MV4-11 (R248W) cells with TP-0903 (20 nM) for 72 hours as measured by surface expression of CD11b by flow cytometry.***$p < 0.001$, ****$p < 0.0001$.

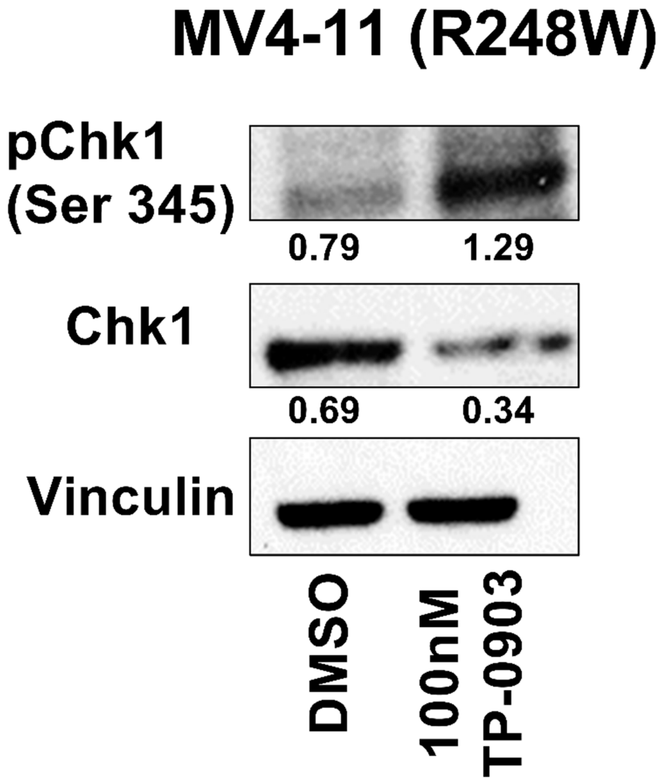

Figure S4. Effects of TP-0903 on pChk1 in MV4-11 *TP53* mutant (R248W) cells. MV4-11 (R248W) cells were treated with 100 nM TP-0903 for 4 hours. Western blot analysis was performed on whole-cell lysates run on parallel gels with the indicated antibodies. Vinculin served as the loading control for each lysate. Immunoblots were quantified against respective loading controls using ImageJ.

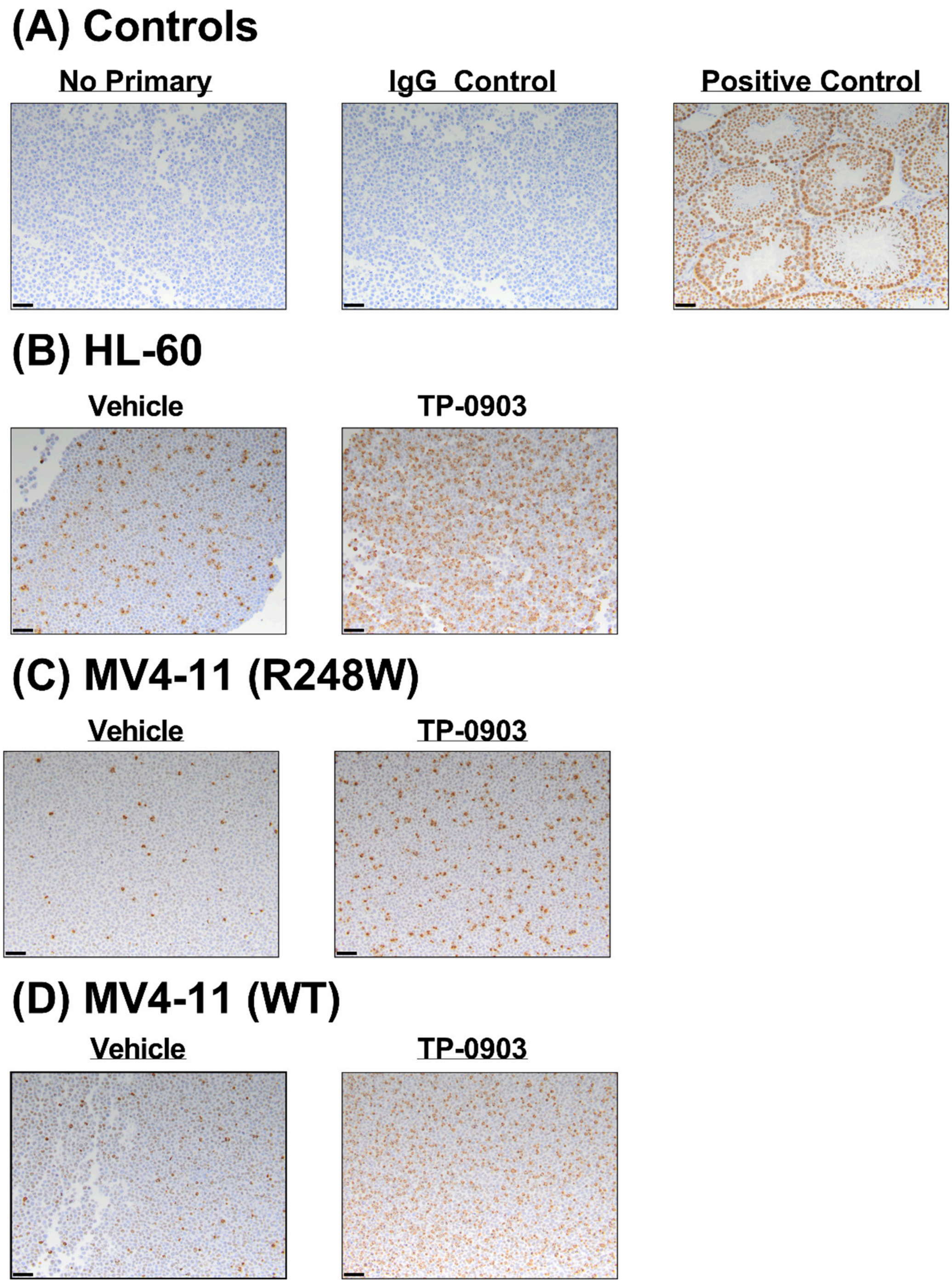

Figure S5. Immunohistochemistry demonstrating upregulation of pH2AX with TP-0903 treatment. **(A)** pH2AX immunohistochemistry was optimized using no primary, IgG, and positive controls. **(B)** HL-60, **(C)** MV4-11 *TP53* mutant (R248W), or **(D)** MV4-11 wild-type (WT) cells were treated with DMSO or 100nM TP-0903 for 4 hours, prepared for immunohistochemistry and stained for pH2AX. Each image was taken at 200X total magnifications. The scale bar represents 50 micrometers.

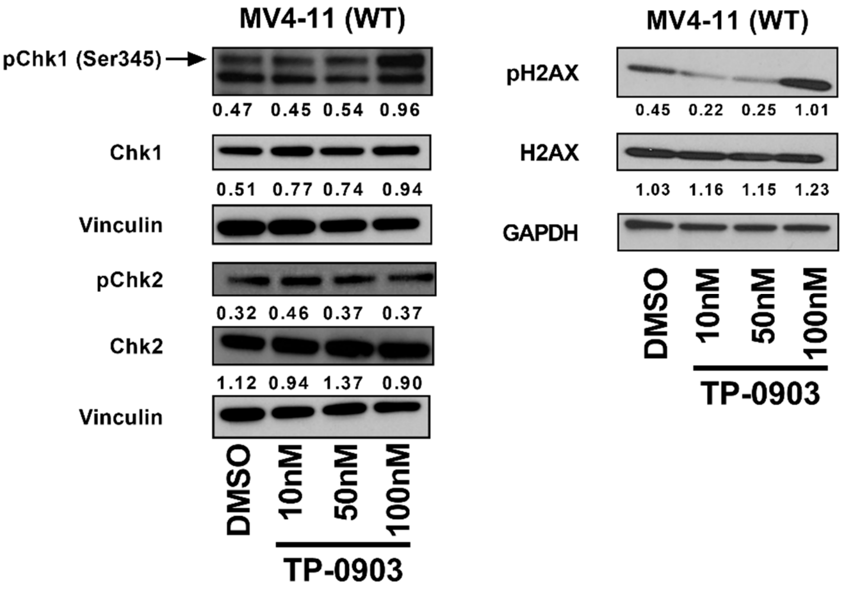

Figure S6. TP-0903 upregulates pChk1 kinase and pH2AX in MV4-11 cells. MV4-11 wild-type (WT) cells were treated with DMSO or increasing concentrations of TP-0903 for 4 hours. Western blot analysis was performed on whole-cell lysates run on parallel gels with the indicated antibodies. Vinculin or GAPDH served as the loading control for each lysate. Immunoblots were quantified against respective loading controls using ImageJ. Data are representative of 1-2 independent experiments.

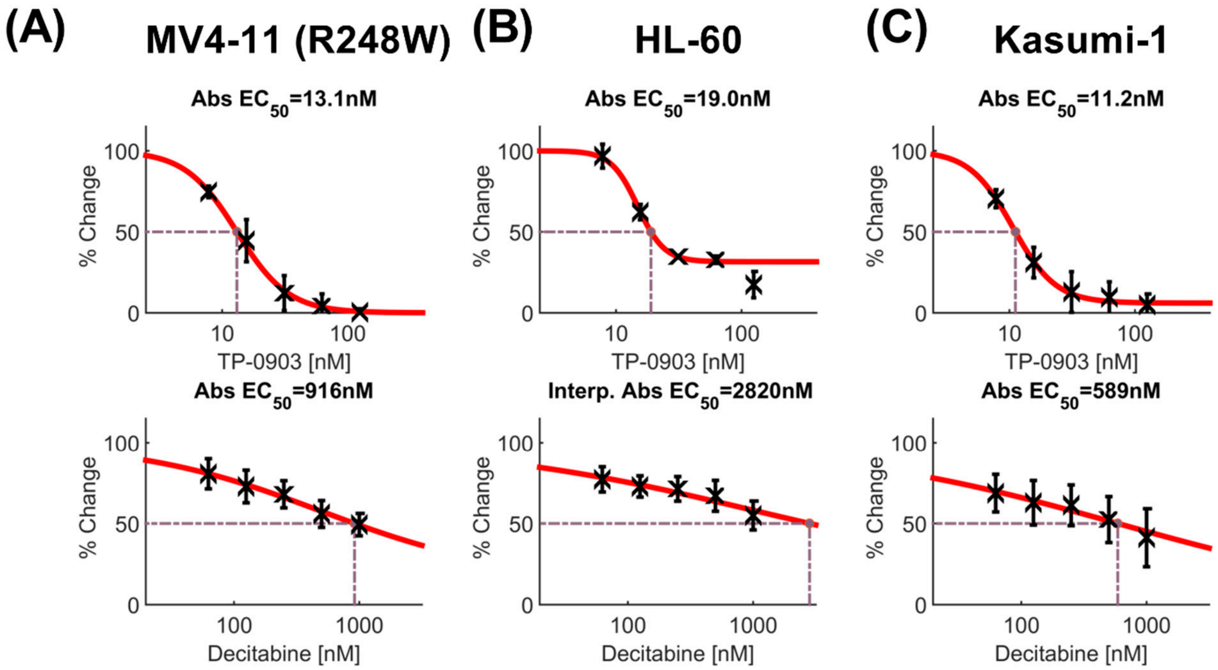

Figure S7. In vitro activity of TP-0903 or decitabine in combinatorial assays. MTT cell viability assays were performed by treating **(A)** MV4-11 (R248W), **(B)** HL-60, and **(C)** Kasumi-1 cell lines with varying concentrations of TP-0903 and decitabine for 72h (n = 18 across three independent experiments).
